# Synthetic studies towards bottromycin

**DOI:** 10.3762/bjoc.8.189

**Published:** 2012-10-01

**Authors:** Stefanie Ackermann, Hans-Georg Lerchen, Dieter Häbich, Angelika Ullrich, Uli Kazmaier

**Affiliations:** 1 Institute of Organic Chemistry, Saarland University, P.O. Box 151150, 66041 Saarbrücken, Germany; 2 Bayer Pharma Aktiengesellschaft, Aprather Weg 18a, 42113 Wuppertal, Germany

**Keywords:** amidines, antibiotics, bottromycin, peptides, thiopeptides, Ugi reactions

## Abstract

Thio-Ugi reactions are described as an excellent synthetic tool for the synthesis of sterically highly hindered endothiopeptides. *S*-Methylation and subsequent amidine formation can be carried out in an inter- as well as in an intramolecular fashion. The intramolecular approach allows the synthesis of the bottromycin ring system in a straightforward manner.

## Introduction

Natural products are excellent sources as lead structures for the development of new antibiotics. Over millions of years microorganisms, such as bacteria and fungi, developed efficient defense strategies against their bacterial competitors [1–3]. Not surprising, a wide range of common antibiotics such as penicillin or vancomycin are natural products or derivatives thereof.

**Figure 1:**
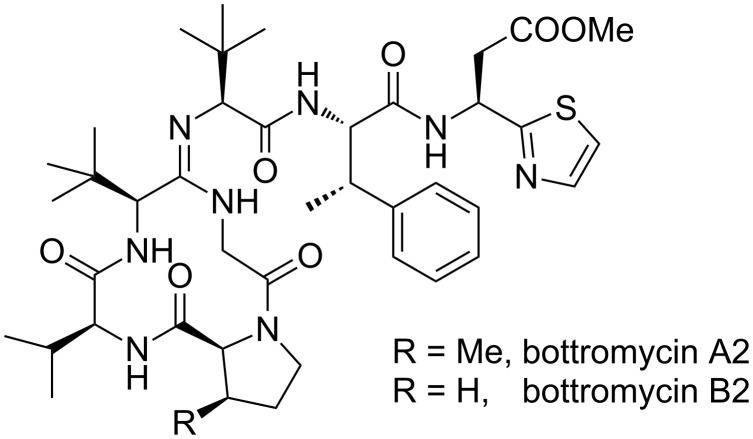
Bottromycins.

In 1957 Waiswisz et al. reported a new antibiotic peptide isolated from the fermentation broth of *Streptomyces bottropensis*, called bottromycin [4–6]. This antibiotic inhibits the growth of a wide range of microorganisms by interfering with their protein biosynthesis [7–12]. In 1965 Nakamura et al. isolated closely related antibiotics from the strain *Streptomyces* No. 3668-L2, named bottromycin A and B [13–14]. Acidic hydrolysis provided a mixture of all-(*S*)-configured amino acids containing 3-methylphenylalanine [15], *tert*-leucine, valine, β-(2-thiazolyl)-β-alanine [16] and glycine. While also proline was found in bottromycin B, *cis*-3-methylproline is a component in bottromycin A [17]. Originally Nakamura postulated a linear *N*-acylated iminohexapeptide structure, a proposal which was revised after synthetic studies [18] as well as NMR spectroscopic investigations by Takita et al., which proposed a cyclic iminopeptide structure [19]. This proposal was verified by Schipper [20] and Kaneda [21], based on detailed NMR studies. According to them, the bottromycins are cyclic tetrapeptides, connected to a tripeptidic side chain through an amidine structure. The different bottromycins differ only in the substitution pattern of the proline (Figure 1). The three-dimensional structure was reported recently by Gouda et al. [22]. This structure is quite unusual, not only because of the amidine moiety but also because most amino acids are found in β-methylated form (*tert*-leucine can be seen as β-methylvaline).

Although a series of synthetic studies towards linear bottromycin sequences have been published [23–24], no total synthesis was reported for a long time. The first and only synthesis so far was described by Sunazuka and Ōmura et al. in 2009 [25]. Their synthesis was based on the formation of the amidine structure by reaction of the tripeptide side chain with an endothiopeptide and ring closure between proline and glycine. The same group also undertook some modifications on the natural product [26].

## Results and Discussion

Our group is also involved in the synthesis of peptide-based natural products [27–32], and of course the structure of bottromycin is highly fascinating from a synthetic point of view. In our previous investigations we observed that for the synthesis of sterically demanding peptides, especially those containing *N*-alkylated amide bonds, Ugi reactions are especially suited [33–34]. For *N*-unsubstituted peptides the Ugi reactions should be carried out with ammonia as the amine component, which is a protocol that is often accompanied by a range of side reactions. But in general the yields are good if sterically demanding aldehydes, such as pivaldehyde are used [35–36]. With thiocarboxylic acids as acid components, this approach allows also the synthesis of endothiopeptides [37–40], and therefore this protocol should be extremely suitable for the synthesis of bottromycins.

**Scheme 1:**
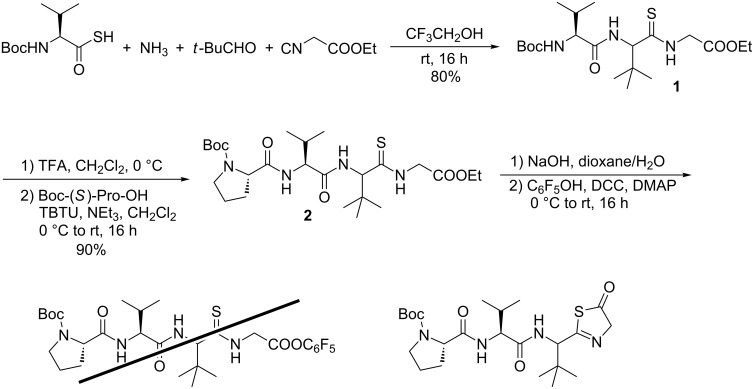
Syntheses of endothiopeptides by thio-Ugi reaction.

To prove this option, we reacted Boc-protected (*S*)-thiovaline [41] with pivaldehyde, ethyl isocyanoacetate, and a 2 M NH_3_ solution in CH_3_OH. In trifluoroethanol, which is the best solvent for ammonia Ugi reactions [35], the expected endothiopeptide **1** was formed in high yield as a 1:1 diastereomeric mixture (Scheme 1). With this building block in hand, we were interested to see whether we would be able to generate the required cyclic endothiopeptide and if we could even subsequently connect the side chain.

Therefore, we prolonged the peptide chain **2** under standard peptide coupling conditions and tried to activate the linear peptide chain. We chose the pentafluorophenylester protocol developed by Schmidt et al. for cyclization [42]. But unfortunately this approach failed (as did all other activations investigated) because of the formation of thiazolone side product, which could not be cyclized.

**Scheme 2:**
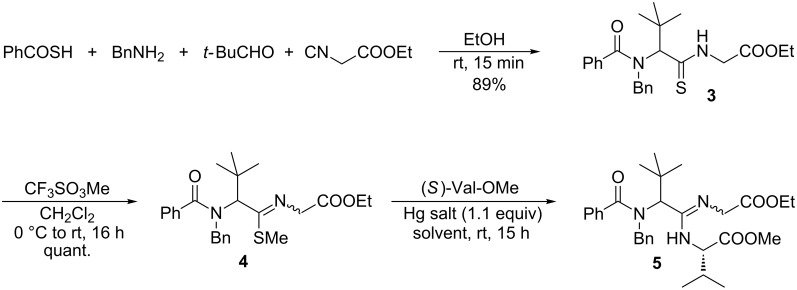
Synthesis of amidine **5** by thio-Ugi reaction.

In parallel, we tried to figure out if the amidine formation is possible with sterically hindered endothiopeptides (Scheme 2). As a model substrate we chose thiopeptide **3** [37], easily obtained by thio-Ugi reaction in excellent yield. The reaction was very fast and was finished already after 15 min, and peptide **3** crystallized directly from the reaction mixture. Because our first attempts to couple **3** directly with amines to the corresponding amidine **5** failed [43], we decided to convert **3** into the corresponding thioimidate **4**, which was reacted with (*S*)-valine methyl ester as an amine component in the presence of Hg salts [44]. Hg(OCOCF_3_)_2_ was found to be more suitable than Hg(OCOCH_3_)_2_ (Table 1, entries 1 and 2) and from the different solvents tested, THF was the solvent of choice. With a twofold excess of the amine component a yield of up to 72% of **5** could be obtained as a 1:1 diastereomeric mixture. The diastereomers could be separated by flash chromatography, but unfortunately these reaction conditions could not be transferred to the thioimidoester obtained from endothiopeptide **2**. Here, the expected amidine was only formed in a trace amount, and a range of side products was obtained.

**Table 1:** Optimization of amidine formation.

entry	Hg salt	equiv (*S*)-Val-OMe	solvent	yield (%)

1	Hg(OCOCH_3_)_2_	1.3	MeCN	0
2	Hg(OCOCF_3_)_2_	1.3	MeCN	24
3	Hg(OCOCF_3_)_2_	1.3	CH_2_Cl_2_	50
4	Hg(OCOCF_3_)_2_	1.3	THF	61
5	Hg(OCOCF_3_)_2_	2.0	THF	72

**Scheme 3:**
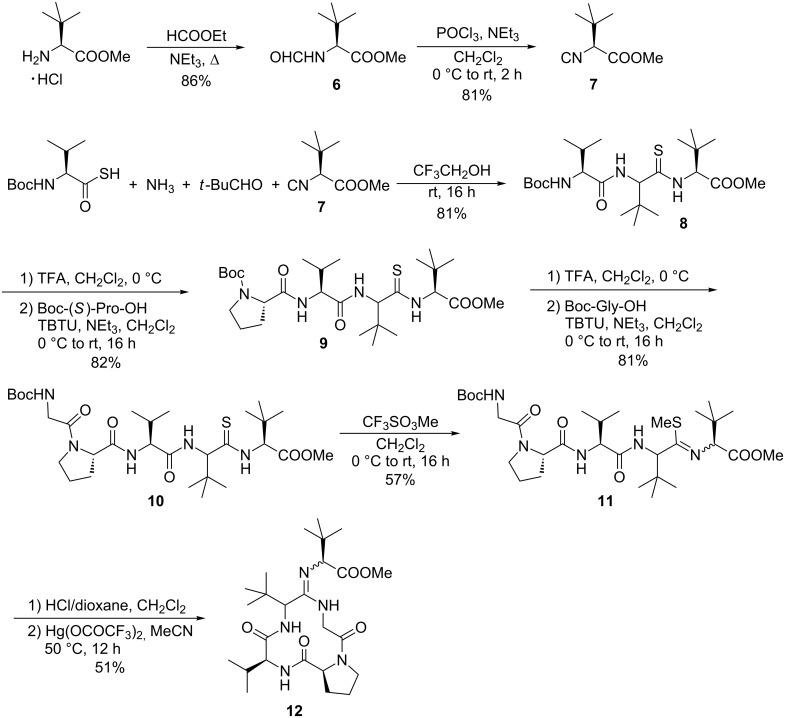
Synthesis of the bottromycin ring system **12** by thio-Ugi reaction.

Therefore, we decided to change our strategy and to replace the intermolecular amidine formation by an intramolecular one. In this case the peptide ring should be formed in the amidination step. For this approach we first needed the isocyanide **7** derived from (*S*)-*tert*-leucine methyl ester. According to Ugi et al. this isocyanide was obtained in enantiomerically pure form from the corresponding formamide **6** by dehydration with POCl_3_/NEt_3_ (Scheme 3).

This isocyanide was subjected to a thio-Ugi reaction as described before, and the expected sterically highly demanding endothiopeptide **8** was obtained in high yield as a 1:1 diastereomeric mixture. In this case, the diastereomers could not be separated. Elongation of the peptide chain under standard peptide coupling conditions provided the linear precursor **10** for the ring-closing amidination. After removal of the Boc-protecting group the resulting salt showed a low solubility in THF, and therefore we had to run the amidination in CH_3_CN, although this was not the solvent of choice in our model system.

The amidination was carried out under high-dilution conditions. The peptide salt was dissolved in CH_3_CN and was added slowly through a syringe to a solution of Hg(OCOCF_3_)_2_ in CH_3_CN at 50 °C over a period of 10 h. Interestingly, the best result was obtained not with the free peptide amine but with the hydrochloride salt. Here the cyclic amidine was obtained in 51% yield.

## Conclusion

In conclusion, we could show that thio-Ugi reactions are an excellent synthetic tool for the synthesis of highly sterically hindered endothiopeptides. *S*-Methylation and subsequent amidine formations can be carried out in an inter- as well as in an intramolecular fashion. The intramolecular approach allows the synthesis of the bottromycin ring system in a straightforward manner. The synthesis of the bottromycins and derivatives thereof is currently under investigation.

## Supporting Information

File 1 Detailed experimental procedures, NMR and analytical data of all compounds.
