# Supplementary material for: Synthetic studies towards bottromycin
Source: Beilstein J Org Chem. 2012 Oct 1;8:1652–6. doi: 10.3762/bjoc.8.189 (PMC3510998; doi:10.3762/bjoc.8.189)
Supplement: File 1 — Detailed experimental procedures, NMR and analytical data of all compounds. [file Beilstein_J_Org_Chem-08-1652-s001.pdf]

Supporting Information

for

Synthetic studies towards bottromycin

Stefanie Ackermann[1], Hans-Georg Lerchen[2], Dieter Häbich[2], Angelika Ullrich[1] and Uli Kazmaier*[1]

Address: [1]Institute of Organic Chemistry, Saarland University, P.O. Box 151150, 66041 Saarbrücken, Germany and [2]Bayer Pharma Aktiengesellschaft, Aprather Weg 18a, 42113 Wuppertal, Germany

Email: Uli Kazmaier - u.kazmaier@mx.uni-saarland.de

*Corresponding author

Detailed experimental procedures, NMR and analytical data of all compounds

Experimental section

General Information

All reactions were carried out in oven-dried glassware (100 °C) under nitrogen unless otherwise stated. Septa, disposable syringes and needles were used for the transfer of reagents and other liquid chemicals. For the drying of organic phases, water-free sodium sulfate was used.

^{1}H NMR-spectra were measured on a 400 MHz (AVII-400) or a 500 MHz (DRX-500) NMR spectrometer from Bruker. $CDCl_3$ was used as solvent. The solvent peak was calibrated at 7.26 ppm. The analysis of the spectra was done with PC-software MestRe-C or Topspin 3.0. The abbreviations used for interpretation of NMR spectra are: s = singlet, d = doublet, dd = doublet of doublet, t = triplet, q = quartet, m = multiplet or br = broad. Chemical shifts were δ values and were measured in ppm.

^{13}C NMR-spectra were measured at a frequency of 100 MHz (AVII-400) or at 125 MHz (DRX-500) on NMR spectrometers from Bruker. $CDCl_3$ was used as solvent. The solvent peak was calibrated at 77.0 ppm. The analysis of the spectra was done with PC-software MestRe-C or Topspin 3.0. Chemical shifts were δ values and were measured in ppm.

Preparative flash column chromatography was performed by using columns packed with silica gel grade 60 (35–70 µm) purchased from Macherey–Nagel.

Melting points were measured in open glass capillaries on apparatus MEL-TEMP II purchased from Laboratory Devices and are uncorrected.

Optical rotations were measured on a Perkin–Elmer polarimeter PE 341 at 20 °C and λ = 589 nm.

Thin-layer chromatography was done by using commercially available precoated Polygram® SIL-G/UV 254 plates purchased from Fluka. The detection of spots was done under UV-light, I_2 vapours or $KMnO_4$ solution.

Elemental analyses were performed at the Institute for Organic Chemistry, Saarland University, on the instrument Leco (model CHN900).

High-resolution mass spectrometry (HRMS) was performed at the Institute for Organic Chemistry, Saarland University, on a MAT 95Q (Finnigan). The fragmentation was carried out through chemical ionization (CI).

Solvents were dried by heating the corresponding solvent under reflux over a suitable drying agent. Tetrahydrofuran (THF) was dried over lithium aluminium hydride (LAH); dichloromethane (DCM) was dried over powdered CaH_2. Commercial grade solvents such as ethyl acetate, hexane, diethyl ether were distilled prior to use.

Ethyl [{*N*-*tert*-butyloxycarbonyl-(*S*)-valyl-amino}-(*S*/*R*)-3,3-dimethyl-butanethioamido]acetate (1)

Pivaldehyde (603 mg, 7.00 mmol) was added to a 2 M solution of NH_3 in CH_3OH (3.50 mL, 7.00 mmol) at 0 °C. The solution was stirred for 15 min before ethyl isocyanoacetate (792 mg, 7.00 mmol) and Boc-(*S*)-thiovaline (1.52 g, 7.00 mmol) dissolved in CF_3CH_2OH (7 mL each) were added. After being stirred overnight at rt, the mixture was diluted with CH_2Cl_2 (30 mL) and was washed twice with sat $NaHCO_3$ and 1 N $KHSO_4$ solution (10 mL each). After drying (Na_2SO_4) and evaporation of the solvent, the crude product was purified by flash chromatography (silica, hexanes/EtOAc 7:3) to give **1** as a yellow oil (2.44 g, 5.70 mmol, 80%) and a 1:1 mixture of diastereomers. R_f (hexanes/EtOAc = 6:4): 0.17.

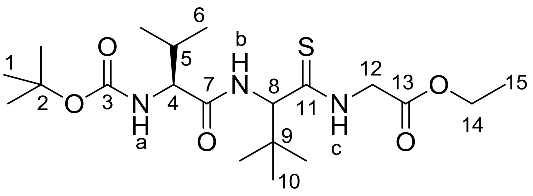

^{1}H NMR (500 MHz): δ = 0.87 (m, 6 H, 6-H), 0.97 (m, 9 H, 10-H), 1.26 (m, 3 H, 15-H), 1.41 (s, 9 H, 1-H), 2.10 (m, 0.5 H, 5-H), 2.19 (m, 0.5 H, 5-H), 4.04 (m, 0.5 H, 4-H), 4.09 (m, 0.5 H, 4-H), 4.12 (dd, $^2J_{12,12}$ = 18.5 Hz, $^3J_{12,NHc}$ = 6.8 Hz, 1 H, 12-H), 4.20 (m, 2 H, 14-H), 4.55 (dd, $^2J_{12,12}$ = 18.5 Hz, $^3J_{12,NHc}$ = 5.5 Hz, 1 H, 12-H), 4.84 (d, $^3J_{8,NHb}$ = 9.5 Hz, 0.5 H, 8-H), 4.87 (d, $^3J_{8,NHb}$ = 9.5 Hz, 0.5 H, 8-H), 5.15 (d, $^3J_{NHa,4}$ = 5.5 Hz, 0.5 H, N-H^a), 5.23 (d, $^3J_{NHa,4}$ = 8.5 Hz, 0. 5H, N-H^a), 7.20 (d, $^3J_{NHb,8}$ = 8.0 Hz, 1 H, N-H^b), 8.84 (bs, 0.5 H, N-H^c), 8.98 (bs, 0.5 H, N-H^c)..

^{13}C NMR (125 MHz): δ = 14.1 and 14.1 (q, C-15), 17.3 and 17.9 (q, C-6), 19.5 and 19.5 (q, C-6), 26.8 (q, C-10), 28.3 and 28.3 (q, C-1), 30.6 (s, C-9), 35.9 and 36.0 (d, C-5), 46.9 and 47.0 (t, C-12), 60.1 and 60.4 (d, C-4), 61.7 and 61.7 (t, C-14), 64.7 and 64.8 (d, C-8), 79.8 (s, C-2), 155.8 (s, C-3), 168.3 and 168.3 (s, C-7), 171.1 and 171.3 (s, C-13), 202.5 and 202.6 (s, C-11).

HRMS (CI) ($C_{20}H_{37}N_3O_5S$) [M$^+$]: calcd 431.2454; found 431.2438.

Ethyl [{*N*-*tert*-butyloxycarbonyl-(*S*)-prolyl-(*S*)-valyl-amino}-(*S*/*R*)-3,3-dimethyl-butanethioamido]acetate (2)

A solution of **1** (868 mg, 2.00 mmol) in CH_2Cl_2 (10 mL) was cooled to 0 °C before trifluoroacetic acid (2 mL) was added. The cooling bath was removed and the mixture was allowed to warm up to rt. The reaction was monitored by TLC. The solvent was evaporated in vacuo. The residue was dissolved in CH_2Cl_2 (10 mL) under argon, and TBTU (706 mg, 2.20 mmol) and Boc-(*S*)-Pro-OH (473 mg, 2.20 mmol) were added.

After being cooled to 0 °C, NEt_3 (1.40 mL, 10 mmol) was added, and the reaction mixture was allowed to warm up to rt overnight. The organic layer was washed with H_2O, sat $NaHCO_3$ and 1 N $KHSO_4$ (10 mL each) and was dried over Na_2SO_4. After evaporation of the solvent in vacuo the crude product was purified by flash chromatography (silica, hexanes/EtOAc 1:1) to give **2** as a colorless oil (954 mg, 1.80 mmol, 90%). R_f (hexanes/EtOAc = 1:1): 0.11.

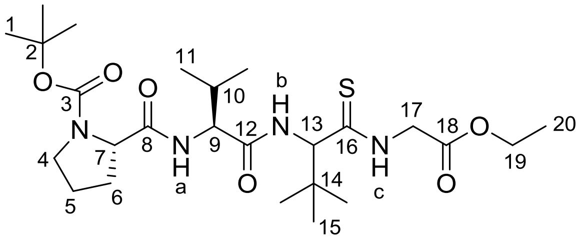

^{1}H NMR (500 MHz): δ = 0.85 (m, 6 H, 11-H), 0.95 (s, 9 H, 15-H), 1.23 (m, 3 H, 20-H), 1.83 (s, 9 H, 1-H), 1.83–2.31 (m, 5 H, 5-H, 6-H, 10-H), 3.35 (m, 2 H, 4-H), 4.14–4.26 (m, 4 H, 17-H, 19-H), 4.30–4.43 (m, 2 H, 7-H, 9-H), 4.67 (d, $^3J_{13,NHb}$ = 9.5 Hz, 1 H, 13-H), 7.14 (bs, 0.5 H, N-H^a), 7.25 (bs, 0.5 H, N-H^a), 7.46 (bs, 0.5 H, N-H^b), 7.60 (bs, 0.5 H, N-H^b), 8.51 (bs, 0.5 H, N-H^c), 8.61 (bs, 0.5 H, N-H^c).

^{13}C NMR (125 MHz): δ = 14.1 (q, C-11), 19.5 (q, C-11), 26.8 (q, C-15), 28.4 (q, C-1), 30.5 (m, C-5, C-6), 35.7 (s, C-14), 38.6 (d, C-10), 46.7 (t, C-4), 46.9 (t, C-17), 58.9 (m, C-7, C-9), 61.7 (t, C-19), 65.5 (d, C-13), 80.4 (s, C-2), 155.3 (s, C-3), 168.3 (m, C-8, C-12), 172.3 (s, C-18), 202.5 (s, C-16).

HRMS (CI): $C_{25}H_{44}N_4O_6S$ [M^+]: calcd 528.2982; found 528.2999.

Ethyl 2-(2-(*N*-benzoyl-*N*-benzylamino)-(*S*/*R*)-3,3-dimethylbutanethioamido)acetate (3)

According to the preparation of **1**, **3** was obtained from thiobenzoic acid (276 mg, 2.0 mmol), benzylamine (215 mg, 2.0 mmol), pivaldehyde (172 mg, 2.0 mmol) and ethyl isocyanoacetate (226 mg, 2.0 mmol) after purification by column chromatography (hexanes/EtOAc 8:2) and recrystallization (PE/EtOAc 3:7) as white needles (760 mg, 1.78 mmol, 89%), mp 144–145 °C. R_f (hexanes/EtOAc = 8:2): 0.16.

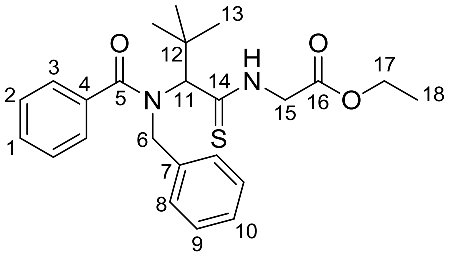

^{1}H NMR (500 MHz): δ = 1.26 (s, 9 H, 9-H), 1.32 (t, J = 7.1 Hz, 3 H, 18-H), 4.22–4.36 (m, 3 H, 15-H, 17-H), 4.34 (d, J = 15.2 Hz, 1 H, 15-H), 4.44–4.60 (m, 2 H, 6-H), 4.84 (bs, 1 H, 11-H), 7.13 (m, 2 H, ArH), 7.23 (m, 3 H, ArH), 7.37–7.52 (5 H, ArH), 11.30 (bs, 1 H, NH).

^{13}C NMR (125 MHz): δ = 14.2 (q, C-18), 29.8 (q, C-13), 36.9 (s, C-12), 47.7 (C-15), 50.7 (t, C-6), 61.5 (t, C-17), 73.1 (d, C-11), 126.8 (C-Ar), 127.9 (C-Ar), 128.5 (C-Ar), 128.8 (C-Ar), 130.1 (C-Ar), 136.7 (C-Ar), 168.3 (s, C-5), 175.4 (s, C-16), 201.4 (s, C-14).

HRMS (CI): $C_{24}H_{30}N_2O_3S$ $[M]^+$: calcd 426.1977; found 426.1971.

Elemental analysis: $C_{24}H_{30}N_2O_3S$ (426.58) calcd C 67.58; H 7.09; N 6.57; found: C 67.45; H 6.89; N 6.87.

Ethyl 2-(2-(*N*-benzoyl-*N*-benzylamino)-(*S*/*R*)-3,3-dimethyl-1-(methylthio)butylideneamino)acetate (4)

Thiopeptide **3** (427 mg, 1.00 mmol) was dissolved in CH_2Cl_2 (4 mL) and the solution was cooled to 0 °C before methyltriflate (125 μl, 1.10 mmol) was added. The reaction mixture was allowed to warm up to rt overnight. NaH (40 mg, 1.00 mmol, 60% in paraffin) was added, and after gas evolution was complete, the solvent was evaporated in vacuo. The crude product was purified by flash chromatography (neutral Al_2O_3, activity grade I, hexanes/EtOAc 8:2) giving rise to **4** as a colorless solid (440 mg, 1.00 mmol, 100%), mp 84–85 °C. R_f (hexanes/EtOAc = 8:2): 0.45.

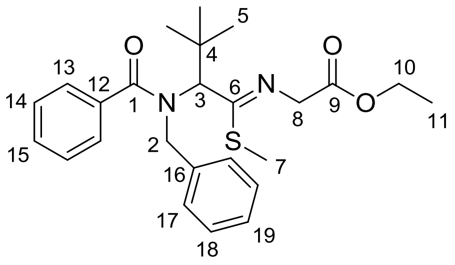

^{1}H NMR (500 MHz): δ = 1.13 (t, $^3J_{11,10}$ = 7.0 Hz, 3 H, 11-H), 1.18 (s, 9 H, 5-H), 2.70 (s, 3 H, 7-H), 3.54 (d, $^2J_{8,8}$ = 18.5 Hz, 1 H, 8-H), 3.96 (d, $^2J_{8,8}$ = 18.5 Hz, 1 H, 8-H), 4.04 (q, $^3J_{10.11}$ = 7.0 Hz, 2 H, 10-H), 4.53 (d, $^2J_{2,2}$ = 16.2 Hz, 1 H, 2-H), 5.28 (d, $^2J_{2,2}$ = 16.2 Hz, 1 H, 2-H), 5.89 (s, 1 H, 3-H), 6.66 (m, 2 H, Ar-H), 6.97 (m, 2 H, Ar-H), 7.09–7.19 (m, 6 H, Ar-H).

^{13}C NMR (125 MHz): δ = 14.1 (q, C-11), 15.7 (q, C-7), 27.8 (q, C-5), 39.1 (s, C-4), 50.5 (t, C-2), 54.3 (t, C-8), 59.2 (d, C-3), 60.5 (t, C-10), 126.0 (d, C-Ar), 126.1 (d, C-Ar), 126.3 (d, C-Ar), 127.8 (d, C-Ar), 128.0 (d, C-Ar), 128.9 (d, C-Ar), 137.6 (s, C-16), 139.8 (s, C-12), 165.0 (s, C-6), 169.9 (s, C-1), 174.2 (s, C-9).

Methyl 2-{[2-(benzoyl-*N*-benzylamino)-*N*-ethoxycarbonylmethyl-(*S*/*R*)-3,3-dimethyl-butyrimidoyl]-amino}-(2*S*)-3-methylbutyrate (5)

Thioimidoester **4** (110 mg, 0.25 mmol) was dissolved in THF (0.5 mL) before (*S*)-Val-OMe (66 mg, 0.5 mmol) was added. Subsequently $Hg(OCOCF_3)_2$ (128 mg, 0.30 mmol) was added and the mixture was stirred overnight at rt. NaH (12 mg, 0.3 mmol, 60% in paraffin) was added, and after gas evolution was finished the solvent was removed in vacuo. The crude product was purified by flash chromatography (neutral Al_2O_3, activity grade I, hexanes/EtOAc 9:1), which allowed separation of the diastereomers. Amidine **5** (96 mg, 0.18 mmol, 72%) was obtained as a colorless solid, mp 105–108 °C (diastereomeric mixture). R_f (hexanes/EtOAc = 9:1): 1. Diastereomer: 0.22; 2. Diastereomer: 0.11.

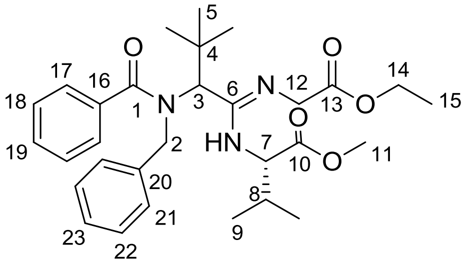

1. Diastereomer:

^{1}H NMR (500 MHz): δ = 0.62 (bs, 3 H, 9-H), 0.80 (bs, 3 H, 9-H), 1.19 (s, 9 H, 5-H), 1.24 (t, $^3J_{15,14}$ = 7.5 Hz, 3 H, 15-H), 1.65 (bs, 1 H, N-H), 2.02 (m, 1 H, 8-H), 3.36 (m, 1 H, 7-H), 3.59 (s, 3 H, 11-H), 3.88 (m, 2 H, 12-H), 4.17 (q, $^3J_{14,15}$ = 7.5 Hz, 2 H, 14-H), 4.30 (m, 1 H, 3-H), 4.49 (m, 2 H, 2-H), 6.71–7.09 (m, 10 H, Ar-H).

^{13}C NMR (125 MHz): δ = 13.2 (q, C-15), 16.8 (q, C-9), 18.7 (q, C-9), 27.3 (q, C-5), 31.4 (d, C-8), 39.8 (t, C-12), 50.6 (q, C-11), 59.9 (m, C-14, C-3), 66.7 (d, C-7), 125.0 (d, C-Ar), 125.9 (d, C-Ar), 126.7 (d, C-Ar), 126.9 (d, C-Ar), 127.5 (d, C-Ar), 132.6 (d,

C-Ar), 138.9 (m, C-16, C-20), 163.9 (s, C-6), 169.0 (s, C-1), 170.7 (s, Ester), 173.7 (s, Ester).

2. Diastereomer:

^{1}H NMR (500 MHz): δ = 0.75 (d, $^3J_{9,8}$ = 6.9 Hz, 3 H, 9-H), 0.86 (bs, $^3J_{9,8}$ = 6.7 Hz, 3 H, 9-H), 1.13 (s, 9 H, 5-H), 1.22 (t, $^3J_{15,14}$ = 7.3 Hz, 3 H, 15-H), 1.75 (bs, 1 H, N-H), 2.02 (m, 1 H, 8-H), 3.35 (m, 1 H, 7-H), 3.58 (s, 3 H, 11-H), 3.89 (m, 2 H, 12-H), 4.14 (q, $^3J_{14,15}$ = 7.3 Hz, 2 H, 14-H), 4.18 (m, 1 H, 3-H), 4.48 (m, 2 H, 2-H), 6.82–7.08 (m, 10 H, Ar-H).

^{13}C NMR (125 MHz): δ = 13.2 (q, C-15), 16.8 (q, C-9), 18.6 (q, C-9), 27.4 (q, C-5), 31.4 (d, C-8), 39.9 (t, C-12), 50.2 (q, C-11), 59.9 (t, C-14), 60.1 (d, C-3), 67.3 (d, C-7), 125.0 (d, C-Ar), 125.8 (d, C-Ar), 126.7 (d, C-Ar), 126.9 (d, C-Ar), 127.5 (d, C-Ar), 132.6 (d, C-Ar), 138.9 (m, C-16, C-20), 163.9 (s, C-6), 169.0 (s, C-1), 170.8 (s, Ester), 173.7 (s, Ester).

HRMS (CI): $C_{30}H_{41}N_3O_5S$ $[M]^+$: calcd 523.3046; found 523.3017.

Methyl (*S*)-*N*-formyl-*tert*-leucinate (6)

A solution of methyl (S)-*tert*-leucinate hydrochloride (14.5 g, 80 mmol) and NEt_3 (12.0 mL, 88 mmol) in ethyl formate (120 mL) was heated under reflux overnight. The solution was filtered through a plug of silica, and after evaporation of the solvent **6** was obtained as a colorless solid (11.9 g, 68.7 mmol, 86%), mp 74–75 °C. R_f (EtOAc): 0.3.

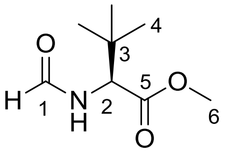

^{1}H NMR (500 MHz) (mixture of rotamers) Major rotamer: δ = 0.92 (s, 9 H, 4-H), 3.68 (s, 3 H, 6-H), 4.50 (d, $^3J_{2,NH}$ = 9.7 Hz, 1 H, 2-H), 6.15 (bs, 1 H, N-H), 8.18 (s, 1 H, 1-H). Minor rotamer (selected signals): δ = 3.69 (s, 3 H, 6-H), 7.91 (d, $^3J_{1,NH}$ = 8.9 Hz, 1 H, 1-H).

^{13}C NMR (125 MHz) (mixture of rotamers) Major rotamer: δ = 26.5 (q, C-4), 34.8 (s, C-3), 51.9 (d, C-2), 58.4 (q, C-6), 160.6 (s, C-1), 170.5 (s, C-5). Minor rotamer: δ = 26.2 (q, C-4), 34.6 (s, C-3), 52.1 (d, C-2), 63.6 (q, C-6), 163.6 (s, C-1), 170.5 (s, C-5).

$[\alpha]^{20}_D$ = +14.2 (*c* 1.0, $CHCl_3$; (*S*), >99% ee)

Elem. Anal. $C_8H_{15}NO_3$ (173.21): calcd C 55.47; H 8.73; N 8.09; found: C 55.48; H 8.54; N 8.07.

HRMS (CI): $C_8H_{15}NO_3$ $[M]^+$: calcd 173.1052; found 173.1070.

Methyl (*S*)-2-isocyano-3,3-dimethylbutyrate (7)

Formamide **6** (10.4 g, 60.0 mmol) and NEt_3 (25 mL, 180 mmol) were dissolved in CH_2CH_2 (100 mL). The solution was cooled to 0 °C before $POCl_3$ (5.51 mL, 60.0 mmol) was added slowly. During 2 h the mixture was warmed to rt and was cooled again to 0 °C before Na_2CO_3 (12.7 g, 120 mmol) in H_2O (400 mL) was added. After being stirred for 1 h at rt, the layers were separated, and the organic layer was washed twice with H_2O (100 mL each), dried (Na_2SO_4) and evaporated in vacuo. The crude product was distilled under reduced pressure (bp_1: 28 °C) to give **7** (7.54 g, 48.6 mmol, 81%) as a colorless oil.

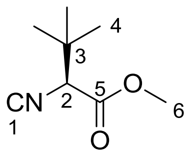

^{1}**H NMR** (500 MHz): δ = 1.03 (s, 9 H, 4-H), 3.74 (s, 3 H, 6-H), 3.92 (s, 1 H, 2-H).

^{13}**C NMR** (125 MHz): δ = 26.2 (q, C-4), 35.2 (s, C-3), 52.8 (q, C-6), 66.4 (dt, $^1J_{2,N}$ = 6.9 Hz, C-2), 160.1 (t, $^1J_{1,N}$ = 1.6 Hz, C-1).

$[\alpha]^{20}_D$: +39.7 (*c* 1.0, $CHCl_3$; (*S*), >99% ee)

HRMS (CI): $C_8H_{13}NO_2$ $[M]^+$: calcd 155.0946; found 155.0996.

Methyl [(*N*-*tert*-butyloxycarbonyl-(*S*)-valinyl-amino)-(*S*/*R*)-3,3-dimethylbutanethioamido]-(*S*)-3,3-dimethylbutyrate (8)

According to the preparation of **1,** thiopeptide **8** was obtained from pivaldehyde (689 mg, 8.00 mmol), Boc-(*S*)-thiovaline (1.87 g, 8.00 mmol) in CF_3CH_2OH (8 mL), isocyanide **7** (1.24 g, 8.00 mmol) and 2 M NH_3 (4.00 mL, 8.00 mmol) in CH_3OH. After workup as described and flash chromatography (hexanes/EtOAc 8:2), **8** (3.05 g, 6.46 mmol, 81%) was obtained as a mixture of diastereomers as a colorless solid, mp. 80–82 °C. R_f (hexanes/EtOAc = 8:2): 0.16.

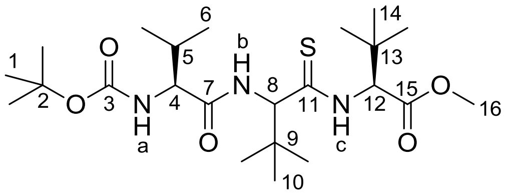

^{1}**H NMR** (400 MHz): δ = 0.83–0.96 (m, 6 H, 6-H), 0.97–1.10 (sh, 18 H, 10-H, 14-H), 1.43 (s, 4.5 H, 1-H), 1.44 (s, 4.5 H, 1-H), 2.09 (m, 0.5 H, 5-H), 2.31 (m, 0.5 H, 5-H), 3.65 (s, 1.5 H, 16-H), 3.73 (s, 1.5 H, 16-H), 3.98 (m, 0.5 H, 4-H), 4.07 (m, 0.5 H, 4-H), 4.85–4-99 (m, 1.5 H, 8-H, 12-H), 5.11–5.21 (m, 1.5 H, 8-H, N-H^a), 7.17 (d, *J* = 8.5 Hz, 0.5 H, N-H^b), 7.30 (d, *J* = 8.7 Hz, 0.5 H, N-H^b), 8.42 (bs, 0.5 H, N-H^c), 8.77 (bs, 0.5 H, N-H^c).

^{13}C NMR (100 MHz): δ = 17.0 and 17.9 (q, C-6), 19.3 and 19.5 (q, C-6), 26.7 and 26.8 (q, C-10/14), 27.0 and 27.1 (q, C-10/14), 28.3 and 28.4 (q, C-1), 30.6 and 31.3 (d, C-5), 34.4 and 35.3 (s, C-9/13), 36.4 (s, C-9/13), 51.8 (q, C-16), 59.9 and 60.4 (d, C-4), 64.9 and 65.1 (d, C-8), 65.7 and 67.1 (d, C-12), 79.7 and 79.9 (s, C-2), 155.5 (s, C-3), 169.7 (s, C-7), 171.4 (s, C-15), 203.8 and 203.8 (s, C-11).

Elem. Anal. $C_{23}H_{43}N_3O_5S$ (473.67): calcd C 58.32; H 9.15; N 8.87; found: C 58.47; H 8.96; N 8.76.

HRMS (CI): $C_{23}H_{43}N_3O_5S$ $[M]^+$: calcdcalcd 473.2923; found 473.2939.

Methyl [(*N*-*tert*-butyloxycarbonyl-(*S*)-prolyl-(*S*)-valinyl-amino)-(*S*/*R*)-3,3-dimethylbutanethioamido]-(*S*)-3,3-dimethylbutyrate (9)

Tetrapeptide **9** was obtained as described for the preparation of **2** from **8** (1.89 g, 4.00 mmol) and Boc-(*S*)-Pro-OH (947 mg, 4.40 mmol). Flash chromatography (silica, hexanes/EtOAc 1:1) provided **9** (1.87 g, 3.27 mmol, 82%) as a pale yellow solid, mp 81–83 °C (mixture of diastereomers). R_f (hexanes/EtOAc = 1:1): 0.23.

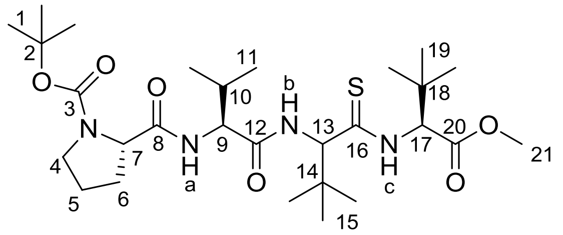

^{1}H NMR (400MHz): δ = 0.84 (m, 6 H, 11-H), 0.93–0.99 (m, 18 H, 15-H, 19-H), 1.39 (s, 9 H, 1-H), 1.81–2.31 (m, 5 H, 5-H, 6-H, 10-H), 3.34 (m, 2 H, 4-H), 3.60 (s, 1.5 H, 21-H), 3.67 (s, 1.5 H, 21-H), 4.25–4.34 (m, 2 H, 13-H, 17-H), 4.82–5.03 (m, 2 H, 7-H, 9-H), 6.88 (bs, 0.5 H, N-H[a]), 7.12 (bs, 0.5 H, N-H[a]), 7.25 (bs, 0.5 H, N-H[b]), 7.52 (bs, 0.5 H, N-H[b]), 8.40 (bs, 0.5 H, N-H[c]), 8.80 (bs, 0.5 H, N-H[c]).

^{13}**C NMR** (100 MHz): δ = 17.4 (q, C-11), 19.5 (q, C-11), 24.7 (d, C-10), 26.7 and 26.8 (q, C-15/19), 27.0 and 27.1 (q, C-15/19), 28.3 and 28.4 (q, C-1), 30.1 (t, C-5/6), 34.5 and 35.1 (s, C-14/18), 36.1 (s, C-14/18), 47.0 (t, C-4), 51.8 (q, C-21), 58.8 (d, C-13), 59.6 (d, C-17), 65.3 and 65.6 (d, C-7), 66.7 (d, C-9), 80.5 (s, C-2), 156.9 (s, C-3), 167.6 (s, C-8/C-12), 167.8 (s, C-8/C-12), 170.4 (s, C-20), 203.1 (s, C-16).

Elem. Anal. $C_{28}H_{50}N_4O_6S$ (570.79): calcd C 58.92; H 8.83; N 9.82; found: C 58.54; H 8.50; N 9.67.

HRMS (CI): $C_{28}H_{50}N_4O_6S$ $[M]^+$: calcd 570.3451; found 570.3420.

Methyl [(*N*-*tert*-butyloxycarbonyl-glycyl-(*S*)-prolyl-(*S*)-valyl-amino)-(*S*/*R*)-3,3-dimethylbutanethioamido]-(*S*)-3,3-dimethylbutyrate (10)

According to the preparation of **2**, tetrapeptide **9** (1.72 g, 3.00 mmol) was deprotected with CF_3COOH (3 mL) in CH_2Cl_2 (15 mL). After workup the crude salt was deprotonated with sat $NaHCO_3$ (2 mL), and the free amine was coupled with Boc-Gly-OH (578 mg, 3.30 mol) and TBTU (1.06 g, 3.30 mmol), NEt_3 (460 μl, 3.30 mmol) in CH_2Cl_2 (15 mL). Flash chromatography (silica, hexanes/EtOAc 3:7) gave rise to **10** (1.53 g, 2.43 mmol, 81%) as a colorless solid, mp 94–96 °C. R_f (hexanes/EtOAc = 3:7): 0.15.

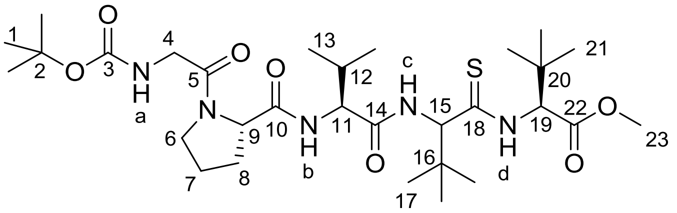

^{1}**H NMR** (400 MHz): δ = 0.81 (m, 6 H, 13-H), 0.87–0.99 (m, 18 H, 17-H, 21-H), 1.37 (s, 9 H, 1-H), 1.85 (m, 2 H, 7-H), 2.09–2.18 (m, 3 H, 8-H, 12-H), 3.39 (m, 1 H, 6-H),

3.50 (m, 1 H, 6-H), 3.61 (s, 1.5 H, 23-H), 3.68 (s, 1.5 H, 23-H), 3.87 (m, 2 H, 4-H), 4.30 (m, 1 H, 15-H), 4.63 (m, 0.5 H, 9-H), 4.84–4.91 (m, 2 H, 9-H, 11-H, 19-H), 5.21 (m, 0.5 H, 11-H), 5.46 (bs, 0.5 H, N-H[a]), 5.64 (bs, 0.5 H, N-H[a]), 7.15 (bs, 1 H, N-H[b]), 7.32 (bs, 0.5 H, N-H[c]), 7.81 (bs, 0.5 H, N-H[c]), 8.12 (bs, 0.5 H, N-H[d]), 9.32 (bs, 0.5 H, N-H[d]).

^{13}C NMR (100 MHz): δ = 17.3 and 17.7 (q, C-13), 19.4 and 19.5 (q, C-13), 24.7 and 24.9 (q, C-17/21), 26.7 and 26.8 (q, C-17/21), 27.1 (q, C-17/21), 28.1 and 28.2 (q, C-1), 30.9 and 31.2 (t, C-7/8), 34.6 and 35.5 (s, C-16/20), 35.9 and 36.2 (s, C-16/20), 43.0 (t, C-4), 46.4 and 46.4 (t, C-6), 51.8 and 51.9 (q, C-23), 58.7 and 58.8 (d, C-11), 59.7 and 60.0 (d, C-9), 64.8 and 65.4 (d, C-15), 65.3 and 66.7 (d, C-19), 79.6 (s, C-2), 155.7(s, C-3), 168.4 (s, C-amide), 170.1 (s, C-amide), 170.9 (s, C-amide), 171.6 (s, C-22), 203.6 (s, C-18).

HRMS (CI): $C_{30}H_{53}N_5O_7S$ $[M]^+$: calcd 627.3666; found 627.3622.

Methyl 2-[{(*N*-*tert*-butyloxycarbonyl-glycyl-(*S*)-prolyl-(*S*)-valyl-amino)-(*S*/*R*)-3,3-dimethyl-1-(methylthio)butylidene}amino]-(*S*)-3,3-dimethylbutyrate (11)

According to the preparation of **4**, pentapeptide **10** (882 mg, 1.40 mmol) was *S*-methylated with methyltriflate (175 µl, 1.55 mmol) in CH_2Cl_2 (5 mL) and NaH (56 mg, 1.40 mmol, 60% in paraffin) to give **11** (513 mg, 0.80 mmol, 57%) as a colorless solid, mp 86–88 °C. R_f (hexanes/EtOAc = 3:7): 0.24.

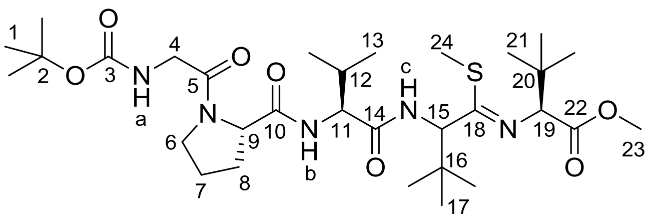

^{1}H NMR (400 MHz): δ = 0.87 (m, 6 H, 13-H), 0.98–1.07 (m, 18 H, 17-H, 21-H), 1.44 (s, 9 H, 1-H), 1.86–2.35 (sh, 5 H, 7-H, 8-H, 12-H), 2.50 (s, 1.5 H, 24-H), 2.57 (s, 1.5 H, 24-H), 3.40 (m, 1 H, 6-H), 3.53 (m, 1 H, 6-H), 3.63 (s, 1.5 H, 23-H), 3.66 (s, 1.5 H, 23-H), 4.09 (m, 2 H, 4-H), 4.15 (s, 1 H, 19-H), 4.23 (dd, $^{3}J_{11,NHb}$ = 8.0 Hz, $^{3}J_{11,12}$ = 5.2 Hz, 0.5 H, 11-H), 4.26 (dd, $^{3}J_{11,NHb}$ = 8.4 Hz, $^{3}J_{11,12}$ = 6.0 Hz, 0.5 H, 11-H), 4.55 (m, 0.5 H, 9-H), 4.63 (m, 0.5 H, 9-H), 4.88 (d, $^{3}J_{15,NHc}$ = 9.6 Hz, 0.5 H, 15-H), 4.93 (d, $^{3}J_{15,NHc}$ = 9.6 Hz, 0.5 H, 15-H), 5.43 (m, 1 H, N-H^{a}), 6.74 (d, $^{3}J_{NHc,15}$ = 9.6 Hz, 0.5 H, N-H^{c}), 6.78 (d, $^{3}J_{NHc,15}$ = 9.6 Hz, 0.5H, N-H^{c}), 7.31 (m, 1 H, N-H^{b}).

^{13}C NMR (100 MHz): δ = 15.8 and 15.9 (q, C-24), 17.6 and 17.7 (q, C-13), 19.3 (q, C-13), 24.9 and 24.9 (t, C-7/8), 26.2 and 26.6 (q, C-17/21), 26.9 and 27.0 (q, C-17/21), 27.4 and 27.5 (q, C-17/21), 28.3 (q, C-1), 30.4 and 31.0 (t, C-7/8), 35.3 and 35.6 (s, C-20), 36.3 and 36.5 (s, C-16), 43.0 (t, C-4), 46.3 (t, C-6), 51.2 and 51.3 (q, C-23), 56.2 and 56.3 (d, C-15), 58.8 and 59.1 (d, C-11), 60.0 and 60.1 (d, C-9), 73.0 and 73.7 (d, C-19), 79.8 (s, C-2), 155.7 (s, C-3), 167.4 and 167.8 (s, C-ester/amide), 169.7 (s, C-ester/amide), 170.4 (s, C-ester/amide), 170.8 and 170.8 (s, C-ester/amide), 170.9 and 171.0 (s, C-ester/amide).

HRMS (CI): $C_{31}H_{55}N_5O_7S$ $[M]^{+}$: calcd 641.3822; found 641.3775.

Methyl 2-[cyclo-{*N*-(glycyl-(*S*)-prolyl-(*S*)-valyl)-2-amino-(*S*/*R*)-3,3-dimethyl-butylidene}-amino]-(*S*)-3,3-dimethyl-butyrate (1*2*)

Peptide **11** (128 mg, 0.20 mmol) was dissolved in CH_2Cl_2 (1 mL), and the solution was cooled to 0 °C before 4 M HCl (0.50 mL, 2.00 mmol) in dioxane was added. The reaction was monitored by TLC, and after complete conversion the solvent was evaporated in vacuo. The oily residue was stirred in Et_2O resulting in the precipitation of the pentapeptide hydrochloride as colorless salt. The ether was decanted and the salt was dried in vacuo. $Hg(OCOCF_3)_2$ (109 mg, 0.25 mmol) was dissolved in MeCN (50 mL) and the solution was heated to 50 °C. The pentapeptide salt (123 mg, 0.20 mmol) was also dissolved in MeCN (2 mL) and the solution was added slowly (0.2 mL/h) via syringe pump to the warm Hg-salt solution. After complete addition stirring was continued for 2 h. The solution was cooled to rt, the precipitate was filtered off and the solvent was evaporated in vacuo. Flash chromatography (basic Al_2O_3, activity grade I, EtOAc/MeOH 98:2) gave rise to **12** (51 mg, 0.10 mmol, 51%) as a yellow oil. R_f (EtOAc/CH_3OH = 95:5): 0.14.

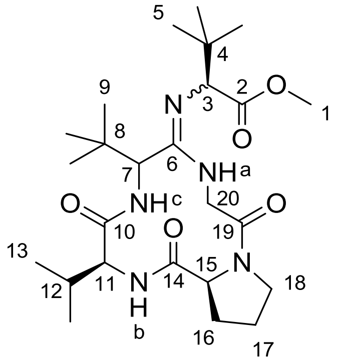

^{1}H NMR (500 MHz): δ = 0.85–1.02 (m, 24 H, 5-H, 9-H, 13-H), 1.81 (m, 0.5 H, 17-H^a), 1.84–1.94 (m, 1.5 H, 17-H^a, 17-H^b), 2.04–2.14 (m, 1.5 H, 12-H, 16-H^a), 2.18 (m, 0.5 H, 12-H), 2.31 (m, 1 H, 16-H^b), 3.55 (m, 2 H, 18-H), 3.68 (s, 1.5 H, 1-H), 3.71 (s, 1.5 H, 1-H), 3.92 (m, 1 H, 20-H^a), 3.96–4.17 (m, 3 H, 3-H, 15-H, 20-H^b), 4.23 (d, $^3J_{11,NHb}$

= 7.0 Hz, 0.5 H, H-11), 4.27 (d, $^3J_{11,NHb}$ = 7.2 Hz, 0.5 H, H-11), 4.35 (d, $^3J_{7,NHc}$ = 6.7 Hz, 0.5 H, H-7), 4.42 (d, $^3J_{7,NHc}$ = 7.4 Hz, 0.5 H, H-7), 4.66 (d, $^3J_{NHa,20b}$ = 5.7 Hz, 1 H, N-H[a]), 6.41 (d, $^3J_{NHc,7}$ = 6.9 Hz, 0.5 H, N-H[c]), 6.45 (d, $^3J_{NHc,7}$ = 7.2 Hz, 0.5 H, N-H[c]), 6.92 (d, $^3J_{NHb,11}$ = 6.9 Hz, 1 H, N-H[b]).

^{13}C NMR (125 MHz): δ = 18.3 and 18.8 (q, C-13), 19.4 and 19.5 (q, C-13), 22.4 (t, C-17), 26.4 and 26.5 (q, C-5/9), 26.7 and 26.8 (q, C-5/9), 29.2 (t, C-16), 29.9 (d, C-12), 34.5 and 34.6 (s, C-4/8), 34.8 and 34.8 (s, C-4/8), 44.0 and 44.1 (t, C-20), 51.8 (t, C-18), 52.6 (d, C-7), 56.8 (q, C-1), 59.9 (d, C-15), 60.3 and 60.4 (d, C-11), 60.9 and 61.1 (d, C-3), 156.8 (s, C-6), 167.5 (s, C-19), 169.0 (s, C-10/14), 170.1 (s, C-10/14), 171.7 and 171.9 (s, C-2).

HRMS (CI): $C_{25}H_{42}N_5O_5$ $[M]^+$: calcd 492.3186; found 492.3156.
